# Natural compounds as multitarget agents in Alzheimer’s diseases: evidence from *in vivo* and *in vitro* models

**DOI:** 10.3389/fphar.2026.1766470

**Published:** 2026-02-23

**Authors:** Vicko Suswidiantoro, Kim San Tang, Khalid Rahman, Donna Maretta Ariestanti, Richard Johari James, Choo Chee Yan, Mitsuyasu Kato, Fadlina Chany Saputri

**Affiliations:** 1 Doctoral Programme, Faculty of Pharmacy, Universitas Indonesia, Depok, West Java, Indonesia; 2 School of Pharmacy, Monash University Malaysia, Bandar Sunway, Selangor, Malaysia; 3 Faculty of Science, School of Pharmacy and Biomolecular Sciences, Liverpool John Moores University, Liverpool, United Kingdom; 4 Faculty of Pharmacy, Universitas Indonesia, Depok, West Java, Indonesia; 5 Integrative Pharmacogenomics Institute (iPROMISE), UiTM Selangor Branch, Bandar Puncak Alam, Selangor, Malaysia; 6 Faculty of Pharmacy, UiTM Selangor Branch, Bandar Puncak Alam, Selangor, Malaysia; 7 Departement of Experimental Pathology, Faculty of Medicine, University of Tsukuba, Tsukuba, Ibaraki, Japan; 8 Laboratory of Pharmacology and Toxicology, Faculty of Pharmacy, Universitas Indonesia, Depok, Indonesia; 9 National Metabolomics Collaborative Research Center, Faculty of Pharmacy, Universitas Indonesia, Depok, West Java, Indonesia

**Keywords:** inflammation, multitarget mechanisms, natural products, neurodegeneration, neuroprotection

## Abstract

**Introduction:**

Alzheimer’s disease (AD), the most common cause of dementia, is marked by a gradual decline in cognitive function driven by amyloid-β (Aβ) deposition, tau hyperphosphorylation, synaptic failure, neuroinflammatory responses, and mitochondrial dysfunction. Despite extensive research efforts, currently available pharmacological treatments provide only limited symptomatic relief and do not prevent disease progression. These shortcomings have fuelled growing interest in natural compounds, which possess pleiotropic biological properties and may more effectively target the multifaceted pathology of AD.

**Methods:**

This systematic review was performed in compliance with the PRISMA 2020 guidelines. Comprehensive literature searches were conducted across PubMed, Scopus, and ScienceDirect to identify preclinical and clinical studies examining the effects of natural compounds in *in vitro* and *in vivo* models relevant to AD. Eligible studies assessed phytochemicals, herbal formulations, marine-derived substances, or nutraceuticals and their impact on core AD-related pathological features.

**Results:**

A total of 41 studies fulfilled the inclusion criteria, including 25 *in vivo* and 16 *in vitro* investigations. Across these studies, natural compounds consistently exhibited neuroprotective effects via multiple mechanisms associated with AD pathogenesis. These included the reduction of oxidative stress and neuroinflammation, inhibition of apoptotic pathways, modulation of amyloidogenic processes, attenuation of Aβ aggregation, regulation of tau-associated signalling, and preservation of synaptic function and cognitive outcomes.

**Conclusion:**

Overall, the available evidence suggests that natural compounds confer multitarget neuroprotective effects that directly engage with key pathological mechanisms underlying AD. Nonetheless, significant translational challenges remain, particularly with respect to bioavailability, compound standardisation, and clinical efficacy. Further robust, well-controlled clinical trials are essential to establish the therapeutic value of these agents as potential disease-modifying interventions for AD.

## 1 Introduction

Alzheimer’s disease (AD) is a slowly progressive neurodegenerative condition and the most common cause of dementia globally (Zhang et al., 2024). Clinically, it manifests as memory loss, cognitive deterioration, and behavioural changes, while its neuropathological hallmarks include extracellular amyloid-β (Aβ) deposition, intracellular neurofibrillary tangles formed by hyperphosphorylated tau, synaptic degeneration, neuroinflammatory processes, and extensive neuronal loss (Huang, 2020; Rahman and Lendel, 2021; Chen et al., 2023). As populations continue to age worldwide, the prevalence of AD is increasing steadily, placing a growing burden on healthcare systems and society at large (Alzheimer’s Assocciation, 2024).

Despite significant advances in elucidating the molecular and cellular mechanisms underlying AD, effective disease-modifying treatments remain unavailable. Currently approved therapies, such as acetylcholinesterase inhibitors and N-methyl-D-aspartate (NMDA) receptor antagonists, offer only modest and transient symptomatic benefits, with little impact on the underlying disease trajectory. More recent therapeutic strategies targeting amyloid pathology have yielded limited clinical success, further highlighting the multifactorial nature of AD, in which amyloid accumulation intersects with oxidative stress, mitochondrial dysfunction, neuroinflammation, disrupted proteostasis, and synaptic impairment (Chin et al., 2022; Chvojkova et al., 2024).

Bioactive molecules from natural sources have consequently gained attention as promising candidates (Lim et al., 2024). In contrast to most synthetic drugs designed for single-target action, natural compounds—including phytochemicals, herbal preparations, nutraceuticals, and marine-derived substances—often exhibit polypharmacology (Khan et al., 2020). This multi-target approach is particularly well-suited to AD, whose pathogenesis involves an intricate web of mechanisms such as oxidative stress, mitochondrial dysfunction, excitotoxicity, protein misfolding, neuroinflammation, synaptic failure, and impaired autophagy (Chibuye et al., 2024; Kumar et al., 2026; Sharma et al., 2021; Yoon, 2025). By simultaneously influencing several of these pathways, natural compounds may provide more comprehensive neuroprotection and a greater potential to modify the disease course.

A substantial body of preclinical research has documented the neuroprotective properties of various natural substances. Polyphenols like curcumin, quercetin, and sesamol demonstrate potent antioxidant and anti-inflammatory activities, while also modulating mitochondrial function and protein aggregation (Abu-Elfotuh et al., 2025; Adelakun et al., 2024; Ali et al., 2022). Other phytochemicals, including alkaloids and terpenoids, can regulate neurotransmitter systems, control apoptotic cell death, and shield neurons from excitotoxic damage (Lim et al., 2020; Nigdelioglu Dolanbay et al., 2021; Tang et al., 2022). Marine-derived agents such as astaxanthin, fucoidan, and omega-3 fatty acids contribute to neuronal membrane stability, dampen inflammatory cascades, and promote neurotrophic signalling (De Cillis et al., 2025; Wen et al., 2025; Zhang et al., 2022; Zhu et al., 2024). Nutraceuticals and functional foods are also increasingly studied, not only for their biological efficacy but also for their accessibility, long-term tolerability, and favourable safety profiles in elderly patients (Puri et al., 2022; Xu et al., 2025).

Furthermore, natural products are being explored as adjuncts to conventional therapies, where they may potentiate benefits or mitigate drug-induced side effects through synergistic actions (Nasim et al., 2022). However, despite encouraging results from laboratory models, clinical application remains limited. Human trials are often too small, infrequent, or methodologically inconsistent to draw firm conclusions about therapeutic efficacy.

Given the expanding literature in this field, a systematic and critical assessment of the evidence is necessary. Such an evaluation can clarify the pharmacological profiles of these compounds, identify existing research gaps, and provide insight into future directions for clinical development. This review, therefore, aims to synthesise findings from preclinical and clinical studies on phytochemicals, herbal medicines, marine-derived substances, and nutraceuticals with reported neuroprotective potential. We will focus on their mechanisms of action, the models used to test them, and their relevance for developing effective strategies to prevent and manage neurodegenerative diseases.

## 2 Materials and methods

### 2.1 Search strategy

The present systematic review was conducted in accordance with the PRISMA 2020 guidelines to ensure transparency and methodological rigor. A comprehensive literature search strategy was developed to identify original research articles investigating the neuroprotective potential of natural compounds in the context of neurodegenerative diseases. The Boolean string applied was: (“Alzheimer’s disease” OR “Alzheimer*” OR “Amyloid-β” OR “tau pathology”) AND (“natural compounds” OR “phytochemicals” OR “plant extract” OR “herbal” OR “botanical” OR “marine-derived” OR “nutraceuticals”). This search string was specifically designed to capture a broad spectrum of studies addressing neurodegeneration while simultaneously narrowing the scope to naturally derived agents. Searches were performed across multiple international databases, including PubMed, Scopus, and ScienceDirect, covering the period from January 2020 to 30 September 2025. To complement the electronic search, the reference lists of included articles and relevant reviews were screened manually to identify additional studies that might have been overlooked during the primary search process. Only articles published in English were considered for inclusion to maintain consistency in the extraction and interpretation of scientific findings.

### 2.2 Methodology for selecting eligible studies

The study selection process was structured and systematic to guarantee objectivity and reproducibility. After compiling records from the databases, we used reference management software to remove duplicates. The screening proceeded in two phases. Initially, two reviewers independently assessed titles and abstracts to identify publications potentially relevant to the neuroprotective activity of natural compounds in neurodegenerative disorders. Articles deemed irrelevant at this stage were excluded. The full texts of the remaining studies were then retrieved and evaluated in detail against predefined eligibility criteria to be included:

• Studies had to investigate naturally derived compounds—such as phytochemicals, herbal preparations, botanical extracts, nutraceuticals, or marine-derived products—in models of AD.
• Employed AD–relevant experimental models (transgenic AD animal models (e.g., APP/PS1, 5xFAD); Aβ- or tau-induced animal models; AD-related cellular models (e.g., Aβ-treated neurons, tau-expressing cells, microglial activation by amyloid).
• Reported outcomes related to AD pathology, such as amyloid accumulation, tau pathology, neuroinflammation, synaptic dysfunction, oxidative stress, neuronal survival, or cognitive performance.

We excluded reports focusing:

• Solely on synthetic molecules, along with review articles, editorials, conference proceedings, and studies lacking pertinent outcome data.

Any disagreements between reviewers were resolved through discussion, with the assistance of a third reviewer consulted if necessary. This process ensured the inclusion of only directly relevant studies of sufficient methodological quality, and the review focuses predominantly on mechanistic and preclinical *in vitro* and *in vivo* evidence rather than clinical intervention outcomes. A PRISMA-compliant flow diagram was created to document the final selection of studies.

### 2.3 Data extraction and quality appraisal

Following the final selection of articles, data were extracted systematically using a pre-designed template to ensure uniformity across studies. The extracted information included bibliographic details (author, year, study location), the type of natural compound investigated, the disease model used (*in vitro* or *in vivo*), and the specific AD condition being studied. Key outcomes—whether molecular, biochemical, behavioural, or histopathological—were recorded alongside the proposed mechanisms for the observed neuroprotective effects. To minimise bias, two reviewers independently performed the data extraction, and any discrepancies were resolved by consensus.

We appraised the methodological quality of the included studies using tools appropriate to each research type. For preclinical animal studies, we employed SYRCLE’s Risk of Bias tool to assess internal validity. The following domains were evaluated: (1) sequence generation, (2) baseline characteristics, (3) allocation concealment, (4) random housing, (5) blinding of caregivers/investigators (performance bias), (6) random outcome assessment, (7) blinding of outcome assessors (detection bias), (8) incomplete outcome data, (9) selective outcome reporting, and (10) other sources of bias. *in vitro* studies were evaluated based on pre-established indicators, including the reproducibility of results, the adequacy of control groups, and the appropriateness of the dose ranges tested. This multi-faceted approach ensured a critical appraisal of the evidence quality prior to synthesis.

### 2.4 Data synthesis and analysis

Due to the heterogeneity in study design, models, and outcome measures, pooling the data quantitatively through meta-analysis was deemed inappropriate. Instead, the findings were synthesised in a qualitative, narrative manner. Studies were organised according to the category of natural compound investigated, the neurodegenerative disease model employed, and the nature of the outcomes assessed. This organisation facilitated the identification of recurring mechanisms of action, including antioxidant activity, anti-inflammatory responses, modulation of apoptosis, and inhibition of protein aggregation. Where relevant, *in vitro* findings were compared with *in vivo* outcomes to highlight consistencies or discrepancies in reported effects. To enhance clarity, summary tables were created to provide an overview of the study characteristics and major results, while figures were used to illustrate the mechanistic pathways implicated across different compounds. This approach facilitated a comprehensive integration of the available evidence, enabling a deeper understanding of the therapeutic promise of natural compounds in neuroprotection and their relevance for the management of AD.

## 3 Results

### 3.1 Study selection

**FIGURE 1:**
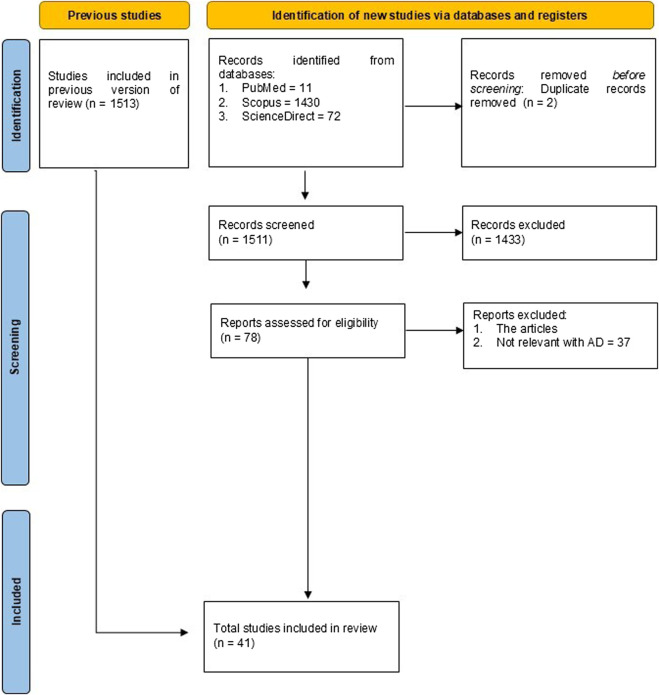
The diagram outlines the identification, screening, eligibility, and inclusion stages of the systematic review.

The literature search yielded a total of 1,513 records from PubMed, Scopus, and ScienceDirect. After duplicate removal, 1,511 titles and abstracts were screened, of which 1,433 did not meet the inclusion criteria and were excluded. The remaining 78 full-text articles underwent detailed evaluation, and 41 studies met all the inclusion criteria for this review. The process of study selection is illustrated in the PRISMA diagram Figure 1.

The final dataset comprised 25 *in vivo* studies Table 1 and 16 *in vitro* studies Table 2. Key methodological details—such as the animal species or cell models used, types of natural compounds investigated, dosing parameters, treatment durations, and assessed endpoints.

**TABLE 1:** Characteristics of the *in vivo* studies included in the systematic review.

Authors	Model/Subjects	Natural compounds extract	Dose/Concentration	Mechanism of action	Key findings
Babaei et al. (2025)	Wistar rats/Male	Epigallocatechin-3-gallate (EGCG)	25, 50, and 100 mg/kg for 3 weeks	↓ BDNF and APP	EGCG has a significant improving effect on cognitive deficits in AD like model rats
Cao et al. (2017)	Wistar rats/Male	Extract methanol *Hypericum perforatum* (HPE)	150, 300 mg/kg 60 days	Reversed accumulation of Aβ, antioxidant, anti-inflammatory	HPE is conferred neuroprotection against AlCl3-induced AD like pathology
Deng et al. (2022)	APP/PS1 mice/Male and Female	*Acorus tatarinowii* volatile oil and total ginsenoside	*Acorus tatarinowii* volatile oil 15–60 mg/kg Total ginsenoside 75–300 mg/kg 30 days	↓ Aβ, AChE levels, LC3B and Beclin-1 expressions ↑ p-Akt, p-mTOR, P62 and Bcl-2 expression	Combination total ginsenoside and *Acorus tatarinowii* volatile oil coadministration may play an autophagy inhibited role in preventing AD development
Gong et al. (2025)	APP/PS1 and C57BL6/J mice/Male	Traditional Chinese medicine prescription Danggui Shaoyao San (DSS)	7.5, 15, 30 g/kg 30 days	Antioxidant, ↑ cognitive function	DSS alleviates AD symptoms by suppressing neuronal ferroptosis via the AMPK/Sp1/ACSL4 axis
Hawiset et al. (2025)	Wistar rats/Male	Tacca chantrieri Andre rhizome extract (TCE)	50, 100, 200 mg/kg 14 days	↓ proinflammatory cytokines, glial fibrillary acidic protein (GFAP) expression; ↑ serotonin	The neuroprotective effects of TCE suggested its potential as a therapeutic agent for memory impairment associated with AD
Jahanshahi et al. (2025)	Wistar rats/Male	Pomegranate seed oil	0.32 and 0.64 mg/kg 2 weeks	↓ Aβ; ↑ neurons in the hippocampus	Pomegranate seed oil demonstrated significant neuroprotective and therapeutic effects by enhancing neuronal density and reducing amyloid plaque formation in the hippocampus
Li et al. (2022)	APP/PS1 and C57BL/6J mice/Male and Female	*Radix Bupleuri*	20, 40, 80 mg/kg 30 days	↓ Aβ, β-secretase 1 (BACE1) expression, NF-κB ↑ expression of Beclin-1 and LC3-II	Total saikosaponins from *Radix Bupleuri lays a* comprehensive role in the treatment of AD through the abovementioned ways, which can effectively ameliorate the cognitive impairment in AD mice
N’Go et al. (2021)	Wistar rats	*Chrysophyllum perpulchrum* extract	300 mg/kg 14 days	Antioxidant	*Chrysophyllum perpulchrum* to counteract full Aß physiopathology mechanisms before promising to be a drug candidate for AD treatment
Nguyen et al. (2025)	5xFAD mice/Male and Female	*Ginkgo biloba* leaves	100 mg/kg 2 months	Anti-inflammatory effects, ↓ Aβ, AChE inhibition	Extract *Ginkgo biloba* associated with a reduction of amyloid plaque load in prefrontal cortex and beneficial effects on markers of neurodegeneration, inflammation, cholinergic signalling and microglial overreactivity
Harquin Simplice et al. (2024)	Wistar rats/Male	Extract Guiera senegalensis (GS)	100, 200, 400 mg/kg 14 days	↑ (ACh), Antioxidant ↓ IL-10, ↓ BDNF levels, ↓ Aβ_1-42_, ↓ phosphorylated Tau, IL-1β, TNF- α, IL-6, IFN-γ, and GFAP levels in hppocampus	Extract of GS holds promise as a potential treatment for AD and exhibited significant anticholinesterase, antioxidant, and anti-inflammatory effects, improving cognitive function while reducing oxidative stress and neuroinflammation
Sun et al. (2020)	Sprague Dawley rats/Male	Seed of *Litchi chinensis* (SLF)	120, 240, 480 mg/kg 28 days	↑Expression level of AKT, ↓GSK-3β and tau	These results indicate that SLF improvescognitive function and prevents hippocampal neuronal injury inrats with Ab25-35-induced AD
Xie et al. (2021)	Sprague Dawley rats/Female	Erzhi pills	0.50, 1.50 g/kg 35 days	↓ Aβ_1-40_, GSK-3β and tau; ↑Bcl-xl, and Bcl-2	Erzhi pills may serve as a potential agent for AD therapeutics by improvinglearning and memory
Onifade et al. (2025)	Male Wistar rats (AD model)	*Persea americana* (PA) and *Tabebuia rosea* (TR)	PA 200 mg/kg, TR 200 mg/kg (14 days)	AChE inhibition, antioxidant	Improved memory, reduced oxidative stress
Rabie et al. (2023)	Wistar Rats (AlCl_3_-induced AD model)	*Callistemon citrinus* extract	200, 400 mg/kg	Antioxidant, anti-AChE, anti-amyloid	Improved memory, reduced amyloid burden
Chheng et al. (2020)	male ICR mice (scopolamine model)	Kleeb Bua Daeng formula	100, 300 mg/kg (1 week)	Antioxidant, anti-amyloid, anti-apoptotic	Improved memory, reduced apoptosis markers
Hu et al. (2020)	APP/PS1 mice	Shexiang Baoxin Pill	22.5, 45 mg/kg (2 months)	Anti-amyloid, anti-inflammatory	Rescued cognitive impairment
Khan et al. (2025)	Mice (AlCl_3_-induced neurotoxicity)	Erqember (polyherbal)	10–20 mL/kg (35 days)	↓AChE, antioxidant, histological protection	Improved learning, reduced anxiety
Mohamed et al. (2024)	Male rats (AlCl_3_-induced AD)	Peanut meal extract	300 mg/kg (5 weeks)	AChE inhibition, antioxidant	Improved oxidative stress, ↑DA, serotonin
Tang et al. (2022)	APP/PS1 mice	Neferine (exosome)	10 mg/kg (15 days)	↑BBB penetration, ↓Aβ load	Reduced AD pathology, improved motor deficits
Yizibula et al. (2021)	Male Wistar rats (Aβ injection AD model)	TCM formula (10 herbs)	1.5, 3, 6 g/kg (21 days)	Antioxidant, ↓Aβ expression	Improved memory, reduced oxidative stress
Zhang et al. (2021)	Male Sprague-Dawley rats (Aβ-induced AD)	Naodesheng tablets	45, 90, 180 mg/kg (2 weeks)	Anti-amyloid, antioxidant	Improved cognition
Sun et al. (2020)	Male Sprague-Dawley rats (Aβ25–35-induced AD)	*Litchi chinensis* seed fraction	120, 240, 480 mg/kg/day (28 days)	AKT/GSK-3β modulation	Improved memory, reduced apoptosis
Kim et al. (2022)	Rats (AD + high-fat diet)	*Forsythiae* Fructus & *Cassiae* Semen	200 mg/kg/day	Insulin signallin, gut microbiota	Prevented memory deficits, reduced Aβ
Tang et al. (2022)	5xFAD mice	OABL	20 mg/kg (3 weeks)	Anti-inflammatory, anti-amyloid	Improved cognition, reduced plaques
Wang et al. (2025)	Male C57BL/6 mice (AlCl_3_-induced AD)	Gallic acid	25, 50, 100 mg/kg (12 weeks)	p38/MAPK inhibition, antioxidant	Reduced neurotoxicity, improved memory

↑: Increased. ↓: Reduced.

**TABLE 2:** Characteristics of the *in vitro* studies included in the systematic review.

Authors	Model/Subjects	Natural compounds extract	Dose/Concentration	Mechanism of action	Key findings
Dibacto et al. (2022)	SK-N-SH cells (H_2_O_2_ stress)	Cameroonian spices (*Xylopia parviflora, Tetrapleura tetraptera, Dichrostachys glomerata, Aframomum daniellii, Zanthoxylum xanthoxyloides, Piper guineense*)	The most effective activity Xylopia parviflora	Anticholinesterase, antioxidant	↑Neuronal survival, ↓cell death
Nigdelioglu Dolanbay et al. (2021)	PC12 cells (H_2_O_2_ damage)	Glaucium corniculatum alkaloids	497 μg/mg	↓ROS, ↑Bcl-2, cell cycle regulation	Suppressed apoptosis, oxidative stress
Tang et al. (2022)	BV-2, PC12 cells	OABL	BV-2 (1, 2.5, 5, 10 µM) PC12 (1, 5.10 µM)	Anti-inflammatory, anti-amyloid	Improved cognition, reduced plaques
Wang et al. (2025)	PC12, HT22, SH-SY5Y	Gallic acid	1, 5, 10, 20, dan 40 µM	p38/MAPK inhibition, antioxidant	Reduced neurotoxicity, improved memory
Wen et al. (2025)	SH-SY5Y	Marine crinoid- derived natural compound (+)-rhodoptilometrin (RDM)	10, 20 *µ*M	Autophagy-mediated protection	Restored locomotion, protected neurons
Ojo et al. (2024)	Enzyme assays	*Ptaeroxylon obliquum, Bauhinia bowkeri*	80, 160 μg/mL	AChE, BuChE, BACE-1 inhibition	Prevented Aβ aggregation
De Torre et al. (2022)	AChE assays	*Origanum vulgare* extract	62.5, 125, 250 μg/mL	AChE inhibition	Strongest inhibition vs. galantamine
Iwuanyanwu et al. (2023)	BV-2, HT-22, hCMEC	Multiple extract	50 μg/mL	Anti-inflammatory, antioxidant	Prevented neurotoxicity, BBB damage
Kashyap et al. (2020)	*in vitro* Aβ assays	Scopoletin from *Argyreia speciosa* Roots	10, 20, 40 µM	Antiamyloid, AChE and BuChE inhibitor	Inhibited Aβ fibrillation
Kpemissi et al. (2023)	*in vitro* + *ex vivo* brain tissue	*Combretum micranthum*	25–400 μg/mL	Antioxidant, anti-AChE	Strong neuroprotection
Lim et al. (2020)	BV-2 and HT22 cells	*Crinum asiaticum* extract	12.5, 25, 50 μg/mL	AChE inhibition, ↓oxidative stress	Protected neurons
Ureña-Vacas et al. (2022)	SH-SY5Y (H_2_O_2_ stress)	*Dactylina arctica* *Nephromopsis stracheyi* *Tuckermannopsis americana* and *Vulpicida pinastri*	Dactylina arctica (10 μg/mL) Nephromopsis stracheyi (25 μg/mL) Tuckermannopsis americana (50 μg/mL) and Vulpicida pinastri (5 μg/mL)	Antioxidant, mitochondrial protection	Reduced oxidative damage
Wang et al. (2024a)	SH-SY5Y	Formononetin	2.5, 5, 10 µM	Nrf2 activation	Protected DA neurons
Wang et al. (2024b)	SH-SY5Y	Curculigo capitulata compounds	5, 10, 20, 40 µM	Nrf2/HO-1 activation	Identified novel neuroprotectives
Tan et al. (2022)	PC12 cells (H_2_O_2_ stress)	Gastrodia elata polyphenols	50, 100, 200 μg/mL	Antioxidant, anti-apoptotic	Protected against oxidative stress
Zhang et al. (2022)	SH-SY5Y	Eremophilanes from marine sponge-associated *Penicillium copticola* fungus	Neuroprotection (5, 10, 20, 30, 40 µM) Antioxidant activity (10, 20, 50, 100 µM)	Antioxidant and neuroprotective	Neuroprotection

### 3.2 Characteristics of included studies

#### 3.2.1 Experimental models

**FIGURE 2:**
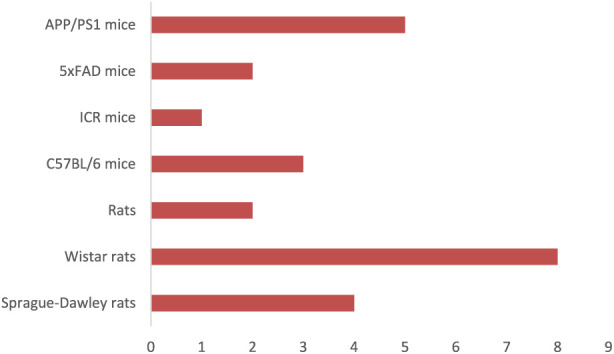
Experimental Animals Applied in *in vivo* Research. The chart compares how often different animal models—such as rats, mice (various strains)—are used across the evaluated studies. Each bar represents the number of studies employing that specific model.

**FIGURE 3:**
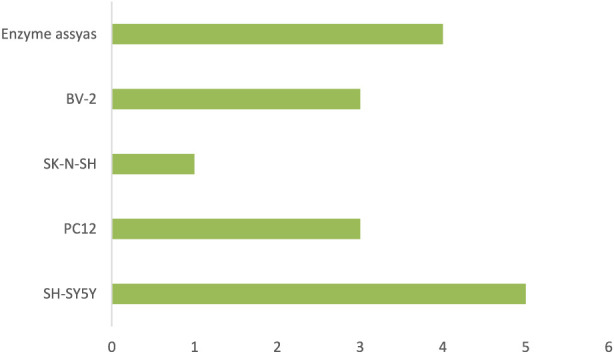
Cell Lines Employed in *In vitro* Experiments. The chart illustrates how often various cell lines—such as SH-SY5Y, PC12, HT22, BV-2, and enzyme assays—were employed across the analyzed studies.

The studies employed a wide array of experimental platforms that collectively represent the major pathological processes underlying AD. These approaches enabled the evaluation of oxidative injury, inflammatory signalling, protein misfolding, mitochondrial impairment, excitotoxicity, and neurotransmission deficits in complementary ways. Characteristics models are illustrated in Figures 2, 3.

Rodent models commonly used included:

1. Wistar and Sprague–Dawley rats
2. Swiss albino and C57BL/6 mice
3. Transgenic strains that replicate AD/PD pathology (e.g., APP/PS1, 5xFAD, LRRK2-G2019S)

*In vitro* systems included:

1. Neuronal cell lines such as SH-SY5Y, PC12, and HT22
2. Microglial and glial cultures, particularly BV-2 cells.
3. Primary neuronal cultures harvested from the cortex or hippocampus
4. Biochemical enzyme assays evaluating acetylcholinesterase (AChE)/butryrylcholinesterase (BChE) inhibition and β-secretase activity
5. Assay platforms for Aβ fibrillation, reactive oxygen species (ROS) quantification, and mitochondrial performance

Together, these models provided a comprehensive framework to investigate cellular and molecular mechanisms relevant to neurodegenerative disorders.

#### 3.2.2 Categories of natural compounds

The 41 eligible studies examined a broad spectrum of naturally derived substances originating from plants, marine organisms, and dietary sources. Variations in dosing strategies, treatment length, and delivery methods contributed to methodological heterogeneity. Compounds examined across studies included:

1. Polyphenols: curcumin, quercetin, sesamol, gallic acid, resveratrol
2. Terpenoids and related derivatives: daphnetin, formononetin, OABL
3. Alkaloids: purified alkaloids and complex alkaloid-enriched extracts
4. Flavonoids and phenolic glycosides
5. Traditional herbal mixtures: Xiaoyao San, Naodesheng tablets, Shexiang Baoxin
6. Marine-derived substances: rhodoptilometrin, astaxanthin, butanolides
7. Nutraceutical or functional food components: peanut meal extract, lichen derivatives, *Moringa* spp., *Persea americana.*

#### 3.2.3 Neuroprotective outcomes across studies

Overall, the included studies demonstrated that natural compounds exhibit a wide range of neuroprotective activities, often acting through multiple pathways simultaneously.

#### 3.2.4 Antioxidant and redox-regulating effects

Robust antioxidant actions were consistently reported across both cellular and animal models. Notable findings related to oxidative regulation included:

1. Marked reductions in intracellular ROS, nitric oxide (NO), and superoxide levels
2. Lower concentrations of oxidative stress markers such as malondialdehyde, protein carbonyls, and oxidised DNA
3. Restoration of key antioxidant enzymes
4. Increased intracellular glutathione (GSH) levels
5. Stabilised mitochondrial membrane potential and reduced mitochondrial swelling
6. Activation of the nuclear factor erythroid 2-related factor 2 (Nrf2) pathway and upregulation of ARE-linked antioxidant genes (HO-1, NQO1, GCLC). More than four-fifths of the studies reported measurable improvements in redox homeostasis.

#### 3.2.5 Anti-inflammatory activity

A substantial number of studies recorded strong anti-inflammatory actions, especially in models triggered by toxins, LPS, or amyloid accumulation. Reported anti-inflammatory mechanisms included:

1. Inhibition of NF-κB signalling pathways
2. Lower expression of pro-inflammatory cytokines (TNF-α, IL-1β, IL-6)
3. Reduced inducible nitric oxide synthase (iNOS)
4. Decreased activation of microglia in hippocampal, cortical, and striatal areas
5. Suppression of LPS-induced NO generation in BV-2 cells
6. Enhanced IL-10 and other anti-inflammatory mediators
7. Preservation of supportive glial phenotypes.

Polyphenols and marine-derived agents consistently demonstrated broad anti-inflammatory activity across multiple inflammatory cascades.

#### 3.2.6 Anti-apoptotic effects and neuronal survival

Across diverse injury paradigms, natural compounds have been shown to significantly attenuate apoptosis and promote neuronal survival. Key anti-apoptotic outcomes included:

1. Elevated Bcl-2 levels
2. Reduced Bax expression and diminished cytochrome c release
3. Lower activation of caspases-3, -8, and -9
4. Preservation of mitochondrial integrity
5. Decreased TUNEL-positive cells in neuronal tissues
6. Activation of pro-survival signalling pathways

These molecular responses often corresponded with improvements in behavioural performance in rodent studies.

#### 3.2.7 Modulation of amyloidogenic processing and Aβ aggregation

Multiple studies reported that natural compounds interfered with amyloid pathology through:

1. Reduction of Aβ plaque burden in transgenic AD mice
2. Inhibition of Aβ fibril formation and oligomerization *in vitro*
3. Downregulation of BACE1 expression or activity
4. Activation of autophagic processes, including AMPKα phosphorylation, ULK1 activation, mTOR inhibition, and. SIRT1-mediated deacetylation

Together, these findings indicate a consistent ability of natural agents to promote proteostasis and reduce pathological protein accumulation.

#### 3.2.8 Neurotransmitter regulation and synaptic protection

Natural compounds have also been shown to influence neurotransmission and synaptic integrity across several disease models. Cholinergic system:

1. Inhibition of AChE and BChE
2. Increased acetylcholine availability
3. Upregulation of choline acetyltransferase (ChAT) expression in the hippocampus

Serotonergic system:

1. Modulation of 5-HT1B receptor signalling
2. Enhanced serotonin metabolism or turnover in specific models

Synaptic integrity:

1. Reduced synaptic degeneration in AD-related models

Some multi-herbal formulations also influenced neurotransmission indirectly by modulating the gut–brain axis,thereby restoring microbial profiles associated with improved neurochemical balance.

## 4 Results

### 4.1 Antioxidant mechanisms

Oxidative imbalance is a hallmark early event in the pathology of AD. The accumulation of ROS drives mitochondrial dysfunction (Wen et al., 2025), lipid peroxidation, and ultimately, neuronal death (Zheng et al., 2024). Many compounds in this review exhibited potent antioxidant properties. For instance, curcumin, especially in nanoformulated versions, activated the Nrf2 signalling pathway, bolstered endogenous antioxidant defenses, and lowered markers of lipid peroxidation like malondialdehyde (Ali et al., 2022). Extract methanol *Hypericum perforatum* (HPE) (150, 300 mg/kg) 60 days, elevating levels of superoxide dismutase (SOD), catalase, and GSH (Cao et al., 2017). Other agents, including quercetin (30 mg/kg for 35 days), peanut meal extract, and various polyherbal preparations, consistently mitigated oxidative stress and supported redox homeostasis (Adelakun et al., 2024; Khan et al., 2025; Mohamed et al., 2024).

Cellular assays yielded parallel findings. Alkaloids from *Glaucium corniculatum* reduced ROS in PC12 cells exposed to hydrogen peroxide (497 μg/mg) (Nigdelioglu Dolanbay et al., 2021), while extracts of *Xylopia parviflora* protected neuronal cultures through a combination of antioxidant and anticholinesterase activities (Dibacto et al., 2022). Marine-origin compounds, such as (+)-rhodoptilometrin (10–20 µM) and certain eremophilanes, provided further evidence for their antioxidant (10–100 µM) and neuroprotective properties (Wen et al., 2025; Zhang et al., 2022). Collectively, these results affirm that enhancing antioxidant capacity is a central mechanism by which natural products confer neuroprotective effects, thereby limiting oxidative damage and preserving neuronal viability.

Emerging evidence from diverse experimental systems indicates that antioxidant actions are not incidental benefits but rather represent a core neuroprotective strategy shared by many natural molecules. Several studies further demonstrate that these compounds influence key redox-sensitive transcriptional regulators—such as Nrf2, FOXO, and PGC-1α—triggering a wider network of cellular defenses that extends far beyond the direct neutralisation of ROS (Klotz et al., 2015; Lennicke and Cochemé, 2021). Through these regulatory pathways, natural agents help promote mitochondrial renewal, stabilise electron-transport processes, and support ATP synthesis (Chen et al., 2023), all of which are essential to maintaining neuronal function under stress. Restoring redox equilibrium also appears to dampen a series of harmful downstream events, including aberrant protein aggregation, inflammatory activation, and apoptosis (Duong et al., 2024; Li et al., 2025). This suggests that antioxidant activity forms the basis upon which other protective mechanisms can operate. Taken together, these interrelated effects underscore the potential of natural compounds to serve as truly multitarget interventions capable of modulating several critical steps in the AD process.

### 4.2 Anti-inflammatory properties

Neuroinflammation is another pivotal driver of neurodegeneration, characterised by activated glial cells releasing cytokines such as TNF-α and IL-6, which exacerbate neuronal injury (Zhang et al., 2023). The reviewed natural products consistently demonstrated an ability to dampen these inflammatory responses. For instance by Hawiset et al. (2025) reported thet *Tacca chantrieri* Andre rhizome extract (50–200 mg/kg) for 14 days significantly reduced inflammatory markers, while *Ginkgo biloba* leaves (100 mg/kg administered for 2 months), similarly alleviated hippocampal inflammation (Nguyen et al., 2025). These *in vivo* findings were further supported by *in vitro* studies. The concentration 1–10 µM OABL reduced inflammatory signalling in microglial cells, and extracts of *Crinum asiaticum* showed comparable anti-inflammatory effects (Lim et al., 2020; Tang et al., 2022). The collective evidence strongly suggests that the modulation of neuroinflammation—often working in concert with antioxidant activity—is a defining characteristic of many natural neuroprotective agents.

In addition, the reproducibility of these anti-inflammatory effects across different experimental systems underscores the importance of neuroimmune regulation to the overall activity of natural compounds. Rather than simply blocking pro-inflammatory cytokines, many of these substances seem to influence glial behavior more broadly, nudging microglia and astrocytes back toward a balanced and supportive state (Gonfa et al., 2023). This includes reducing excessive microglial activation, maintaining astrocytic functions that protect neurons, and boosting neurotrophic pathways that help stabilize synapses (Choi et al., 2011). Several compounds have also been shown to interfere with early signalling events—such as TLR4 engagement, MAPK pathway activation, and the assembly of the NLRP3 inflammasome—suggesting that they act upstream in the inflammatory cascade (Paul et al., 2024; Zhao et al., 2024). This layered mode of action is especially meaningful in neurodegenerative conditions, where persistent inflammation fuels oxidative stress, mitochondrial impairment, and protein misfolding in a reinforcing cycle. Interrupting these interconnected processes at multiple points may allow natural anti-inflammatory agents to deliver more sustained neuroprotection and ultimately slow the progression of neuronal damage.

### 4.3 Regulation of apoptosis and neuronal survival

Programmed cell death is a major contributor to neuronal loss in degenerative disorders. Several studies indicated that natural products can modulate apoptotic cascades (Moujalled et al., 2021). Quercetin, for instance, increased the expression of the anti-apoptotic protein Bcl-2 and suppressed pro-apoptotic signalling, thereby reducing neuronal death (Moujalled et al., 2021). Extract *Guiera senegalensis* (GS) (100, 200, 400 mg/kg) 14 days decreased BDNF levels in the hippocampus. Other compounds, including daphnetin (5–160 mg/kg), administered 48 h post-insult, and formononetin 300 μM, enhanced neuronal survival via activation of the Nrf2 pathway and related protective mechanisms (Havasi Mehr et al., 2024; Wang et al., 2024a).

*In vitro* experiments supported these findings: The concentration of 50–200 μg/mL *Gastrodia elata* polyphenols reduced apoptosis in oxidatively stressed neurons, scopoletin inhibited Aβ-associated toxicity, and CAPE promoted neuronal differentiation while upregulating BDNF in both fly using 0.5% concentration and mammalian systems 10, 12 mg/kg (7 days). These outcomes suggest that regulating apoptotic pathways, combined with activating of survival signals such as BDNF and TrkB, is integral to the protective effects of natural compounds (Konar et al., 2020; Tan et al., 2022). In addition, the collective evidence from these studies shows that natural compounds do far more than simply inhibit apoptotic pathways—they appear to shift the neuronal environment toward one that favors survival and recovery.

AD is strongly influenced by dysregulated programmed cell death, a process that often arises from chronic oxidative burden, mitochondrial disruption, and sustained inflammatory activity. Natural compounds counter these stressors by engaging key survival pathways—including BDNF/TrkB, PI3K/Akt, and Nrf2—which collectively support mitochondrial integrity, protect synaptic function, and maintain balanced intracellular calcium signalling. Through the BDNF/TrkB pathway, these molecules can enhance BDNF levels or promote TrkB phosphorylation, subsequently activating MAPK/ERK and PI3K/Akt signalling. This cascade enhances CREB activity, elevates anti-apoptotic proteins such as those from the Bcl-2 family, and reinforces synaptic structure (Lei et al., 2024; Wang Y. et al., 2024).

In the PI3K/Akt system, the phosphorylation of Akt suppresses key pro-apoptotic factors—including Bad, caspase-9, and GSK-3β—while stimulating mTOR-linked pathways that sustain cellular metabolism and survival (Hossini et al., 2016; Tsuruta et al., 2002). These actions help maintain mitochondrial membrane potential and prevent cytochrome c leakage, a hallmark of intrinsic apoptosis.

Activation of the Nrf2/Keap1 pathway allows Nrf2 to escape Keap1 repression and accumulate in the nucleus, where it binds to ARE sequences and drives the expression of antioxidant defences such as HO-1, NQO1, SOD, GPx, and components of glutathione synthesis (Baird and Yamamoto, 2020; Bellezza et al., 2018). This comprehensive antioxidant response reduces ROS buildup and blocks oxidative stress–driven apoptosis. Many natural agents also fine-tune autophagy—often through AMPK stimulation, mTOR inhibition, or SIRT1-mediated deacetylation—facilitating the removal of damaged mitochondria and aggregated proteins that would otherwise provoke apoptotic signalling (Baeken, 2024; Ge et al., 2022; Liu et al., 2024) By maintaining proteostasis, these compounds help suppress ER stress and prevent downstream cell death pathways.

### 4.4 Inhibition of protein aggregation

The accumulation of misfolded proteins—such as amyloid-β plaques, tau neurofibrillary tangles, and α-synuclein inclusions—represents a defining hallmark of various neurodegenerative diseases. A range of natural compounds has demonstrated the ability to interfere with these pathogenic processes. Extracts from *Callistemon citrinus* 200, 400 mg/kg and Naodesheng tablets (45, 90, 180 mg/kg) 2 weeks have been shown to lessen amyloid deposition and improve behavioural performance in animal models (Rabie et al., 2023; Zhang et al., 2021). Likewise, neferine delivered through exosomes successfully crossed the blood–brain barrier, lowered amyloid burden, and enhanced memory outcomes in transgenic mice 10 mg/kg intravenously for 15 days (Tang et al., 2022). The marine-derived molecule (+)-rhodoptilometrin 250 µM (2–5 days) further facilitated the removal of aggregated proteins by stimulating AMPK-dependent autophagy and suppressing mTOR activity.

At the cellular level, scopoletin and the concentration 250 μg/mL from *Origanum vulgare* extract not only inhibited cholinesterase activity but also prevented the assembly of Aβ fibrils (De Torre et al., 2022). Additional plant-derived agents, including those from *Ptaeroxylon obliquum*, have provided further evidence of blocking enzymes that drive amyloidogenic processing at concentrations of 80 and 160 μg/mL (Ojo et al., 2024).

Collectively, these findings show that natural compounds may directly influence the molecular events underlying disease progression rather than merely alleviating symptoms. Their ability to modulate proteostasis offers an even broader therapeutic window, as pathological protein accumulation not only signifies late-stage degeneration but also contributes to early cellular disturbances.

These agents target multiple steps within the aggregation pathway: preventing misfolding, enhancing enzymatic clearance, and promoting autophagy. Autophagy-boosting compounds typically act via AMPK activation, SIRT1-mediated deacetylation of autophagy regulators (Baeken, 2024; Ge et al., 2022), ULK1 phosphorylation, or inhibition of PI3K/Akt/mTOR signalling, thereby strengthening lysosomal breakdown of toxic aggregates (Chen et al., 2025; Chen and Hurley, 2025). Others support the ubiquitin–proteasome system (UPS) by upregulating ubiquitin ligases and proteasomal components responsible for tagging and degrading misfolded proteins (Li et al., 2022). This is particularly relevant because accumulating evidence indicates that soluble oligomers, rather than fully formed fibrils, are the most harmful species driving synaptic defects and neuronal death. Mechanistic improvements may arise from reducing tau hyperphosphorylation through GSK-3β modulation, lowering amyloidogenic cleavage by inhibiting BACE1 (Khan et al., 2023), or limiting α-synuclein oligomer formation via DJ-1/Nrf2 pathway activation, which stabilises proteins under oxidative stress (Yang et al., 2024).

Furthermore, compounds that activate autophagy may counteract the age-related decline in protein-clearance systems, offering both protective and preventive advantages. Some agents were found to stimulate TFEB, the master regulator of lysosomal biogenesis, resulting in enhanced lysosomal capacity and more effective degradation of aggregated proteins (Yang et al., 2024). Taken together, these observations position natural anti-aggregation molecules as promising candidates for disease-modifying therapies across a spectrum of protein misfolding disorders.

### 4.5 Neurotransmitter modulation

Disruptions in neurotransmitter systems are deeply intertwined with the cognitive, emotional, and motor impairments characteristic of AD. The natural compounds reviewed in this section demonstrate broad regulatory actions on cholinergic, dopaminergic, and serotonergic pathways by influencing key enzymes, receptor signalling, and downstream molecular cascades (Küpeli Akkol et al., 2021; Merghany et al., 2025).

In AD models, extracts from *Persea americana* and *Tabebuia rosea* administered at 200 mg/kg for 14 days suppress acetylcholinesterase activity, thereby increasing synaptic acetylcholine levels. This enhancement of cholinergic transmission further engages the PI3K/Akt–CREB axis, supporting learning processes and synaptic strengthening (Onifade et al., 2025). Peanut meal extract administered orally at 300 mg/kg for 5 weeks elevated dopamine and serotonin concentrations, which subsequently stimulated the cAMP–PKA–CREB pathway, leading to improved memory performance and emotional stability (Mohamed et al., 2024). *Acorus tatarinowii* volatile oil (15–60 mg/kg) and total ginsenoside (75–300 mg/kg), 30 days. Growing evidence also highlights significant serotonergic and gut–brain axis interactions. Emodin-8-O-βD-glucopyranoside at concentration of 100, 200, and 500 µM) stimulated 5-HT1B receptor activity, reducing excitotoxic glutamate release through Gi/Go-mediated suppression of cAMP, thereby providing both neuroprotective and antidepressant effects (Yang et al., 2021).

The diversity of neurotransmitter-modulating effects observed across these studies suggests that natural compounds act in a more integrated manner than single-target pharmaceuticals. By simultaneously affecting cholinergic, dopaminergic, and serotonergic pathways, these agents have the capacity to address overlapping symptoms—cognitive deficits, emotional disturbances, and motor dysfunction—common in many NDDs. Several compounds also regulate neurotransmitter-related enzymes (TH, AADC, ChAT), influence degradative processes (Naoi et al., 2025), and adjust receptor sensitivity through pathways such as mTOR–BDNF, PKC, and CaMKII, which collectively promote synaptic plasticity (Singh et al., 2025; Wang et al., 2024c).

The involvement of the gut–brain axis adds yet another dimension, implying that natural compounds may modulate central neurotransmission indirectly through microbial metabolites, short-chain fatty acids, and immune-mediated signalling. Overall, this multilayered regulatory activity underscores neurotransmitter modulation as a pivotal aspect of the neuroprotective properties of natural products.

### 4.6 Translational barriers and research directions

Although preclinical findings are encouraging, their translation into clinical success remains limited. Human trials are still relatively few, typically involving small participant groups and inconsistent study designs. Many natural compounds also suffer from pharmacological limitations such as low solubility, rapid metabolic degradation, and restricted penetration across the blood–brain barrier (Douroumis and Fahr, 2013; Lestari et al., 2023). Curcumin exemplifies this issue: despite its strong performance in laboratory models, clinical outcomes have been modest due to its poor bioavailability. To overcome these barriers, researchers are investigating nanotechnology-based delivery platforms, co-administration with absorption enhancers (Alshamrani et al., 2022).

A further challenge arises from the wide variability of experimental models used in this field. Rodent studies can provide meaningful biological insights, but still fall short of replicating the full complexity of human AD (Alshamrani et al., 2022). Conversely, simpler organisms such as *Drosophila* and *Caenorhabditis elegans* enable detailed mechanistic studies but offer limited translational relevance (Obafemi et al., 2025; Wang and Zheng, 2022). Establishing standardised protocols for treatment conditions, dosing strategies, and outcome measures will be essential for improving comparability and strengthening the overall evidence base.

In addition to methodological hurdles, the inherent chemical complexity of natural products presents another complication in advancing them to clinical application. Crude extracts often contain numerous active constituents, making it difficult to identify the primary therapeutic molecules and maintain consistent activity between batches. Variability in plant source, environmental conditions, extraction processes, and storage can all significantly alter the chemical composition—and therefore the biological effects—of these preparations (Mungwari et al., 2025). For these reasons, rigorous quality-control frameworks governing purity, potency, and standardization are necessary before these compounds can progress toward regulatory approval.

Another limitation is the limited availability of robust, long-term toxicity and pharmacokinetic evaluations. Although many natural substances have traditional medicinal use, this does not ensure safety when administered at therapeutic doses or among vulnerable populations. Comprehensive assessments of absorption, distribution, metabolism, and excretion (ADME), along with possible interactions with conventional medications, are particularly important for older adults, who often take multiple drugs and may be more susceptible to adverse reactions.

Finally, translating results from controlled experimental conditions into real-world clinical settings introduces additional layers of complexity. Factors such as genetic variability, comorbid health conditions, lifestyle differences, and environmental influences can all affect treatment outcomes. Incorporating natural compounds into personalised-medicine frameworks—potentially through biomarker-based patient selection or genomic profiling—may help refine their therapeutic use and ensure that interventions are directed toward individuals most likely to benefit.

### 4.7 Modulation of nitrosative stress and glutamatergic excitotoxicity

Nitrosative stress and glutamatergic excitotoxicity are closely linked pathological processes that play a major role in synaptic failure and neuronal degeneration in AD (Wang et al., 2021; Wu et al., 2025). Excessive generation of reactive nitrogen species (RNS), particularly NO and peroxynitrite, arises largely from the upregulation of iNOS in chronically activated microglia and astrocytes (Dash et al., 2025; Di Meo et al., 2016). Sustained nitrosative pressure promotes protein nitrosylation, lipid peroxidation, mitochondrial dysfunction, and genomic instability, thereby rendering neurons highly vulnerable to excitotoxic damage (Chatterji et al., 2021; Foster and Stamler, 2004; Pérez de la Lastra et al., 2022). In parallel, impaired regulation of glutamate signalling—characterised by excessive synaptic release and reduced clearance—results in prolonged activation of NMDA and AMPA receptors (Escamilla et al., 2024; Kaur et al., 2019), pathological calcium influx (Kim et al., 2025; Mango and Ledonne, 2023), and activation of downstream neurotoxic cascades.

Across the studies reviewed, many natural compounds demonstrated a clear capacity to attenuate nitrosative stress by suppressing iNOS expression, limiting NO overproduction, and reducing peroxynitrite-mediated injury (Chen et al., 2020; Kim and Lee, 2025). These effects were frequently associated with inhibition of NF-κB signalling, a key molecular link between neuroinflammation and nitrosative damage. By tempering inflammatory activation, natural agents indirectly curtail RNS formation, helping to preserve mitochondrial function and prevent nitrosylation-driven disruption of critical enzymes (Cicala and Morello, 2023; Kim and Lee, 2025). This combined anti-inflammatory and anti-nitrosative action appears particularly relevant in AD, where persistent glial activation sustains a hostile microenvironment around degenerating synapses.

Importantly, reductions in nitrosative stress were often accompanied by protection against glutamatergic excitotoxicity. Excess NO and peroxynitrite are known to potentiate NMDA receptor activity and impair astrocytic glutamate transporters, thereby exacerbating extracellular glutamate accumulation (Sharma et al., 2023). Several natural compounds (curcumin and quercetin) interrupted this vicious cycle by restoring redox balance, stabilising intracellular calcium homeostasis, and supporting mitochondrial ATP production—processes that are essential for efficient glutamate uptake and synaptic regulation (Adelakun et al., 2024; Ali et al., 2022). Through these mechanisms, prolonged NMDA receptor overactivation was restrained, preventing calcium-dependent activation of calpains, nitric oxide synthases, and pro-apoptotic signalling pathways.

Beyond these indirect effects, certain natural (EGCG, resveratrol, lycopene) agents appeared to exert more direct influences on glutamatergic neurotransmission. By modulating intracellular signalling pathways such as PI3K/Akt (Paul et al., 2024), NF-κB, Akt, MAPK, and Wnt, these compounds supported synaptic plasticity while limiting pathological overexcitation (Koval et al., 2025; Sarkar et al., 2009). Restoration of these pathways promotes physiological synaptic function rather than maladaptive excitatory signalling. In addition, several studies reported enhanced expression of neurotrophic factors, notably brain-derived neurotrophic factor (BDNF), which plays a central role in stabilising glutamatergic synapses and facilitating activity-dependent synaptic remodelling (Babaei et al., 2025; Harquin Simplice et al., 2024).

At the cellular level, resistance to glutamate-induced toxicity was closely linked to antioxidant capacity and mitochondrial resilience (Dibacto et al., 2022). Excessive glutamatergic stimulation drives mitochondrial calcium overload (Kim et al., 2025; Mango and Ledonne, 2023) and amplifies oxidative and nitrosative stress, ultimately leading to energetic failure. Natural compounds that activated Nrf2-dependent antioxidant pathways or improved mitochondrial stability were particularly effective in disrupting this cascade, simultaneously reducing redox imbalance and excitotoxic vulnerability (Baird and Yamamoto, 2020; Bellezza et al., 2018). This convergence underscores that protection against excitotoxicity is rarely achieved through isolated receptor antagonism, but rather through broader restoration of cellular homeostasis encompassing redox regulation, metabolic integrity, and inflammatory control. The available evidence indicates that natural compounds exert meaningful neuroprotective effects in AD models by targeting the nitrosative stress–excitotoxicity axis as an integrated pathological network.

### 4.8 Preclinical promise versus clinical reality

Although preclinical research provides strong and consistent evidence for the neuroprotective potential of natural compounds, a considerable translational gap remains between experimental success and clinical implementation. Both *in vitro* and *in vivo* studies have demonstrated that natural products act through multiple neuroprotective mechanisms, including antioxidant, anti-inflammatory, anti-apoptotic, anti-aggregatory, and neurotransmitter-modulating effects, highlighting their promise as multitarget agents for complex neurodegenerative disorders (Echeverry et al., 2025; Nahar et al., 2025). Nevertheless, several interconnected barriers continue to limit their progression into clinically effective therapies for AD.

One major challenge relates to unfavourable pharmacokinetic properties, such as poor bioavailability, rapid metabolism, and insufficient penetration of the blood–brain barrier, as exemplified by compounds like curcumin (Zhou and Hu, 2025). Although innovative delivery strategies, including nanoformulations and lipid-based systems, have shown potential to enhance brain targeting (Liu et al., 2025), their clinical efficacy remains largely unconfirmed. In addition, substantial heterogeneity in preclinical AD models, dosing protocols, and outcome measures complicates the translation of findings to human disease, as existing models do not fully capture the chronic, multifactorial nature of neurodegeneration in ageing populations (Dudal et al., 2022; Loewa et al., 2023).

Importantly, the existing evidence base for natural compounds in AD is heavily skewed towards preclinical research. Although mechanistic studies in cellular and animal models consistently report antioxidant, anti-inflammatory, anti-apoptotic, and anti-aggregatory effects, these promising findings have to be translated into clear clinical benefits. Human studies remain sparse and are often limited by small sample sizes, heterogeneous methodologies, and relatively short follow-up periods, which restrict their ability to evaluate disease-modifying outcomes. Consequently, the current clinical evidence is insufficient to support definitive conclusions regarding therapeutic efficacy, optimal dosing strategies, or long-term safety. This disparity necessitates a cautious interpretation of preclinical findings and underscores the pressing need for well-designed, adequately powered randomised controlled trials before meaningful clinical recommendations can be made.

Further limitations arise from the chemical complexity and variability of natural products, which pose challenges for standardisation, reproducibility, and regulatory approval, particularly when crude extracts or multi-component formulations are employed (Zhu et al., 2022). Finally, the integration of natural compounds into personalised treatment strategies remains underdeveloped, despite growing recognition of the influence of genetic, metabolic, and microbiome-related variability on therapeutic responses (Zhu et al., 2022).

## 5 Conclusion

The body of evidence reviewed here indicates that natural products confer neuroprotection through multiple, often interconnected, mechanisms. By reducing oxidative stress, dampening inflammatory responses, regulating apoptotic pathways, limiting pathological protein aggregation, and re-establishing neurotransmitter homeostasis, these compounds offer a comprehensive approach to addressing AD. While significant challenges remain in translating these findings to the clinic, the overall consistency of preclinical data offers strong justification for progressing towards more stringent clinical investigation.

To close the gap between encouraging experimental results and demonstrable clinical benefits, future research should place emphasis on carefully designed randomised clinical trials incorporating robust and clinically meaningful endpoints. Progress will also rely on advances in formulation and delivery methods to improve bioavailability and ensure effective target engagement, together with rigorous standardisation of natural products to guarantee consistency and reproducibility. Moreover, thorough long-term safety and pharmacokinetic assessments are required to define therapeutic windows. Ultimately, the adoption of biomarker-guided precision medicine strategies may facilitate the identification of patient subgroups most likely to benefit. With such a focused and integrated research strategy, natural products could emerge as valuable additions to future therapeutic approaches for AD conditions.

## Data Availability

The original contributions presented in the study are included in the article/supplementary material, further inquiries can be directed to the corresponding author.
